# Hemoglobin S Promotes Glycemic Dysregulation in a Mouse Model of Human Sickle Cell Disease

**DOI:** 10.1210/endocr/bqaf082

**Published:** 2025-04-29

**Authors:** Joseph Louis Zapater, Seth Thomas Nicholoff, Nadia Saib Sweis, Santosh Lumdas Saraf, Brian Thomas Layden

**Affiliations:** Division of Endocrinology, Diabetes and Metabolism, Department of Medicine, University of Illinois at Chicago, Chicago, IL 60612, USA; Department of Medicine, Jesse Brown VA Medical Center, Chicago, IL 60612, USA; Division of Endocrinology, Diabetes and Metabolism, Department of Medicine, University of Illinois at Chicago, Chicago, IL 60612, USA; Department of Medicine, Internal Medicine Residency, University of Illinois at Chicago, Chicago, IL 60612, USA; Division of Hematology and Oncology, Department of Medicine, University of Illinois at Chicago, Chicago, IL 60612, USA; Division of Endocrinology, Diabetes and Metabolism, Department of Medicine, University of Illinois at Chicago, Chicago, IL 60612, USA; Department of Medicine, Jesse Brown VA Medical Center, Chicago, IL 60612, USA

**Keywords:** type 2 diabetes, glucose metabolism, sickle cell trait, sickle cell disease, sickle cell anemia, insulin dynamics

## Abstract

Hemoglobin S (HbS) presents a challenge to identifying glycemic dysregulation, as changes in red blood cell turnover produce inaccurate hemoglobin A1c (HbA1c) and incongruencies between HbA1c and other glycemic control measures. Concerningly, the prevalence of diabetes in those with HbS is rising, and studies demonstrate that HbS increases the risk of diabetes-related complications. Though a link between the sickle cell variant and HbA1c is reported, the precise mechanisms by which HbS affects glycemic control are unknown. Here, we utilized the Townes mouse model of sickle cell disease (SCD) to analyze the effect of sickle cell trait (SCT) and SCD on glucose homeostasis. We found that chow-fed SCD mice had greater ad libitum and fasting blood glucose than SCT or littermate controls from 8 to 20 weeks of age, along with declining fasting serum insulin with aging, regardless of sex. This was not a result of overt alterations in peripheral glucose or insulin tolerance, gross morphological changes in pancreatic structure, or deposition of iron in pancreatic islets. Furthermore, compared with age- and sex-matched SCT and littermate control mice, we found decreased pancreatic insulin content in 20-week-old SCD male mice and significantly reduced pancreatic islet area and β cell mass in SCD males and females. These findings suggest that having 2 copies of the HbS gene promotes early hyperglycemia and the development of pancreatic β cell dysfunction, which may enhance the risk for diabetes in this cohort.

Hemoglobin A1c (HbA1c) is the primary index utilized to assess glycemic regulation and diagnose and manage diabetes (1). However, utilization of HbA1c can be challenging in individuals with hemoglobinopathies. In sickle cell disease (SCD), the formation of hemoglobin S (HbS) tetramers in the deoxygenated state leads to erythrocyte shape distortion, erythrocyte sickling, and premature hemolysis (2). In turn, this reduces the lifespan of red blood cells and shortens their exposure to circulating glucose, resulting in falsely low HbA1c that is incongruent with fasting and 2-hour glucose levels (3, 4). As a result, patients with HbS are less likely to be diagnosed with diabetes, despite increasing lifespan and prevalence of obesity in this cohort (5-10).

HbS is common worldwide, with 300 and 7.7 million having sickle cell trait (SCT) and SCD, respectively (11, 12). Paralleling the increasing prevalence of diabetes in the general population, recent studies indicate that the prevalence of type 2 diabetes mellitus (T2DM) in those with SCD in the United States is increasing and is similar to that of T2DM in African Americans. Furthermore, the prevalence of T2DM in US Veterans with SCT is greater than in those without SCT, and having HbS increases the risk for T2DM diagnosis among Black people in the United Kingdom (13-15). Furthermore, multiple studies demonstrate that having SCT or SCD increases the risk of developing diabetes-related complications, including nephropathy, retinopathy, stroke, and myocardial infarction (15-18). It is becoming more and more critical to understand glycemic regulation in individuals with HbS.

The above studies raise the question as to how HbS affects glucose homeostasis. In a recent study by Hivert et al (19), through an assessment of genetic variants causing hemoglobinopathies, it was found that sickle cell variant (rs334) was associated with HbA1c. Further, Akinlade et al (20), through an analysis of 30 nondiabetic adults with SCD and 20 age- and sex-matched healthy adults in Nigeria, observed significantly lower fasting insulin levels and HOMA2-β% in adults with SCD after an overnight fast, suggesting impairment of β cell insulin synthesis and/or secretion. Another study by Yavropoulou et al (21) comparing patients with SCD with age- and body mass index–matched healthy individuals in Greece—all of whom had normal oral glucose tolerance testing—observed lower fasting insulin levels and impaired β cell function in the patients with SCD. These latter studies suggest the development of pancreatic β cell dysfunction in patients with SCD that is occurring in the absence of a formal clinical diagnosis of diabetes (20, 21).

Given increasing evidence suggesting a role for HbS in modulating glucose homeostasis, understanding the mechanisms by which this is occurring is necessary to determine how to effectively diagnose, treat, and monitor glycemic dysregulation in individuals with HbS. Here, we utilized the Townes transgenic mouse model of human SCD to analyze the glycemic phenotype of SCT and SCD mice compared with littermate controls that do not contain HbS. Our data suggest a role for HbS in modulating glucose homeostasis by promoting higher blood glucose from an early age and potentially leading to the development of pancreatic β cell dysfunction and lower serum insulin levels with aging.

## Materials and Methods

### Animals

Townes transgenic mice of human SCD (Jackson Laboratory; Bar Harbor, ME, USA; stock #013071) were utilized, which contain human knock-in alleles replacing mouse α-globin and β-globin with human α-globin and β-globin. Mice of the SCD genotype develop sickle cell anemia with hematological and histopathological manifestations paralleling findings in humans (22-25). All mice were housed at the Biological Resources Laboratory of the University of Illinois at Chicago (UIC) under a 12-hour light, 12-hour dark cycle with ad libitum access to a chow diet providing 25% of calories from protein, 17% from fat, and 58% from carbohydrates (Envigo #7012; Madison, WI, USA). Studies were approved by the UIC Animal Care and Use Committee and performed according to the Guide for the Care and Use of Laboratory Animals at UIC.

### Creation and Phenotypic Analyses of SCT and SCD Mice

Male and female Townes mice containing human α-globin along with 1 wild-type human β-globin and 1 sickle human β-globin gene were bred together, creating offspring having either 2 normal human β-globin genes (β^A^β^A^; littermate controls, “WT”), 1 normal and 1 sickle human β-globin gene (β^A^β^S^; sickle cell trait, “SCT”), or 2 sickle human β-globin genes (β^S^β^S^; sickle cell disease, “SCD”). Confirmation of genotype was performed using AccuStart II GelTrack PCR SuperMix (Quantabio; Beverly, MA, USA) using the primers in Table 1.

**Table 1.** Genotyping primers for β^A^β^A^, β^A^β^S^, and β^S^β^S^ mice

Primer pairs (5′ → 3′)	Amplicon size	Genotype		
		β^A^β^A^ (WT)	β^A^β^S^ (SCT)	β^S^β^S^ (SCD)
F: TTGAGCAATGTGGACAGAGAAGG R: GTTTAGCCAGGGACCGTTTCAG	320 bp	X	X	
F: TTGAGCAATGTGGACAGAGAAGG R: AATTCTGGCTTATCGGAGGCAAG	250 bp		X	X

Genotyping was performed using DNA purified from a 1 mm section at the tip of the mouse tail, and each set of primers listed. Genotyping that produced a 320 base pair amplicon signified the presence of only normal human β globin chains (β^A^β^A^; “WT”), whereas the production of only a 250 base pair amplicon signified the presence of only sickle human β globin chains (β^S^β^S^; “SCD”). Genotyping that produced both a 320 and 250 base pair amplicon signified the presence of 1 normal and 1 sickle β globin chain (β^A^β^S^; “SCT”).

Abbreviations: SCD, sickle cell disease; SCT, sickle cell trait; WT, wild type.

**Figure 1.**
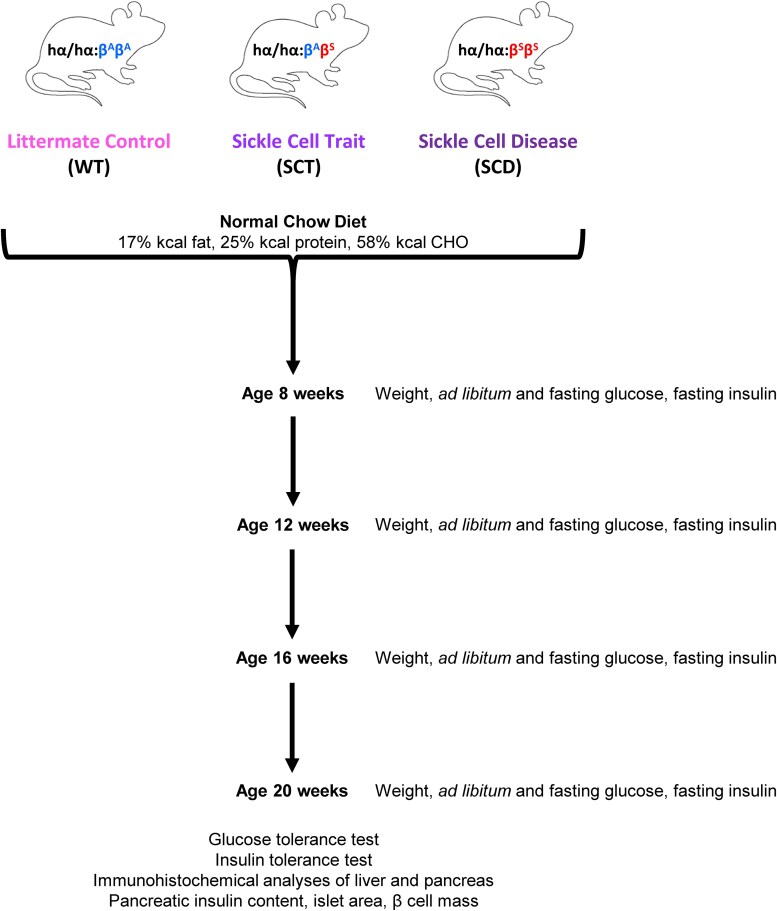
Experimental design. Townes mice containing normal human α globin chains, along with 2 normal copies of the human β globin chain (upper left, Littermate Control, “WT”), 1 normal and 1 sickle human β globin chain (upper middle, Sickle Cell Trait, “SCT”), or 2 copies of the sickle human β globin chain (upper right, Sickle Cell Disease, “SCD”), were fed a normal chow diet and assessed for weight, ad libitum and fasting blood glucose, and fasting serum insulin at 8, 12, 16, and 20 weeks of age. At the age of 20 weeks, glucose and insulin tolerance testing were performed; mice were then euthanized, tissues collected, and immunohistochemical analyses were performed on the liver and pancreas (hematoxylin and eosin, Prussian blue). The pancreas was also analyzed for total insulin content, islet area, and β cell mass.

Mice were assessed for weight, ad libitum and fasting blood glucose, and fasting serum insulin at 8, 12, 16, and 20 weeks of age (Fig. 1). At 20 weeks, glucose and insulin tolerance testing were performed prior to euthanasia and histopathologic analyses.

### Blood Glucose, Serum Insulin, and C-peptide Measurements

Blood glucose was measured at the tail vein using a OneTouch UltraMini glucose monitor (Lifescan Inc; Milpitas, CA, USA). Tail vein blood was collected in heparinized capillary tubes, transferred to Eppendorf tubes, centrifuged at 3500 rpm at 4 °C for 20 minutes, and the supernatant analyzed using an enzyme-linked immunosorbent assay (ELISA) for serum insulin (Crystal Chem; Elk Grove Village, IL, USA; Catalog #90080, RRID:AB_2783626) and c-peptide (Crystal Chem; Elk Grove Village, IL, USA; Catalog #90050, RRID:AB_3678670).

### Glucose and Insulin Tolerance Testing

To assess glucose tolerance, mice were fasted overnight for 16 hours, then administered glucose (2 g/kg body weight) by intraperitoneal injection, and blood glucose and serum insulin were analyzed at various time points as above. To assess insulin tolerance, mice were fasted for 6 hours, then administered 0.75 units/kg body weight of Humalog insulin (Eli Lily & Co.; Indianapolis, IN, USA). Blood glucose was assessed as above.

### Mouse Tissue Collection and Processing

Twenty-week-old mice were euthanized by intraperitoneal injection of ketamine/xylazine followed by cervical dislocation. Tissues were cleaned, weighed, snap frozen, and stored at −80 °C until analysis.

For morphological analyses, pieces of liver and pancreas were placed into cassettes, fixed in 10% formalin for 48 hours, then transferred to 70% ethanol solution. Tissues were embedded in paraffin, sectioned at 5 μM, and transferred to microscope slides by the Histology and Tissue Imaging Core at UIC. For islet and β cell analyses, the mouse pancreas was fixed in 4% paraformaldehyde in 1× phosphate-buffered saline for 1 hour, then incubated at 4 °C overnight in 30% sucrose in phosphate-buffered saline. The pancreas was then cryopreserved in optimal cutting temperature (OCT) compound and sectioned at 7 μm using a microtome-cryostat (Leica Biosystems, Deer Park, IL, USA).

### Immunohistochemistry

Tissue sections were deparaffinized with xylene and rehydrated using solutions containing decreasing ethanol percentages. To analyze pancreatic structure, sections were stained with hematoxylin and eosin using a commercially available staining kit (Vector Laboratories; Burlingame, CA, USA). Tissue iron deposition was analyzed by staining with Prussian blue (Abcam; Waltham, MA, USA). Following staining, slides were mounted with Poly-mount (Polysciences, Inc.; Warrington, PA, USA) and covered with coverslips.

For qualitative morphological assessment of β cells, pancreas sections were deparaffinized and rehydrated as above. Antibody binding sites were unmasked by heat-induced antigen retrieval with sodium citrate buffer (10 mM sodium citrate, 0.05% Tween 20, pH 6.0) using a microwave. Sections were then treated with 3% hydrogen peroxide for 30 minutes to block endogenous peroxidase activity, followed by 0.5% Triton X-100 for 10 minutes for permeabilization. Afterward, sections were blocked with normal goat serum (BioGenex; Fremont, CA, USA), incubated with guinea pig anti-insulin antibody (Agilent Technologies Inc.; Santa Clara, CA, USA; Catalog #IR002, RRID:AB_2800361) diluted in goat serum overnight at 4 °C (1:10 dilution), then with anti–guinea pig IgG peroxidase secondary antibody (Sigma Aldrich; Burlington, MA, USA; Catalog #A5545, RRID:AB_258247) for 1 hour (1:400 dilution in goat serum). Sections were treated with 3,3′-diaminobenzidine substrate (Vector Laboratories; Burlingame, CA, USA; Catalog #SK-4100, RRID:AB_2336382) for 40 seconds and hematoxylin for 20 seconds. Following staining, sections were washed and dehydrated with solutions of increasing ethanol concentration. Following the above staining methods, slides were mounted with Poly-mount (Polysciences, Inc.; Warrington, PA, USA) and covered with coverslips.

All images were acquired using a Leica DMi8 microscope (Leica Biosystems; Deer Park, IL, USA) using a 40× objective.

### Pancreatic Insulin Content

The pancreas tail was homogenized in acid–ethanol (0.2 M HCl in ethanol) and incubated overnight at 4 °C. Homogenates were centrifuged at 2500 rpm at 4 °C for 20 minutes, and insulin in the supernatant was assessed by ELISA. Insulin was normalized to supernatant protein content and assessed using Quick Start Bradford 1× Dye Reagent (Bio-Rad; Hercules, CA, USA).

### Islet Area, Fractional β Cell Area, and β Cell Mass

Mouse pancreas embedded in OCT compound was sectioned as detailed earlier, and 6 nonoverlapping sections spanning the width of each pancreas were used for analysis. Analyses were conducted on 4 mice per genotype. All imaging was obtained using a Leica DMi8 microscope as above, using a 40× objective.

Pancreatic sections in OCT compound were incubated with 3% hydrogen peroxidase, permeabilized with 0.5% Triton X-100, then immunolabeled with guinea pig anti-insulin antibody, anti–guinea pig IgG peroxidase secondary antibody, 3,3′-diaminobenzidine substrate, and hematoxylin as detailed above. Islets were identified on each section and surface area was individually determined using ImageJ software (https://imagej.net/ij/). For determining fractional β cell area, the total pancreatic area and insulin positive area of each section were measured using ImageJ, with the latter represented as a percentage of the total pancreatic area. Utilizing the total pancreatic area and insulin positive area from each section, the β cell mass was estimated as the product of the relative β cell area and the wet weight of the pancreas.

### Statistical Analyses

Statistics were performed using GraphPad Prism and data are presented as means ± SD. Glucose and insulin tolerance tests were analyzed by 2-way analysis of variance (ANOVA) with Bonferroni correction. All other data were analyzed with 2-tailed unpaired Student's *t*-test.

## Results

### Mice With and Without HbS Display Comparable Weight Trends Longitudinally With Aging

**Figure 2.**
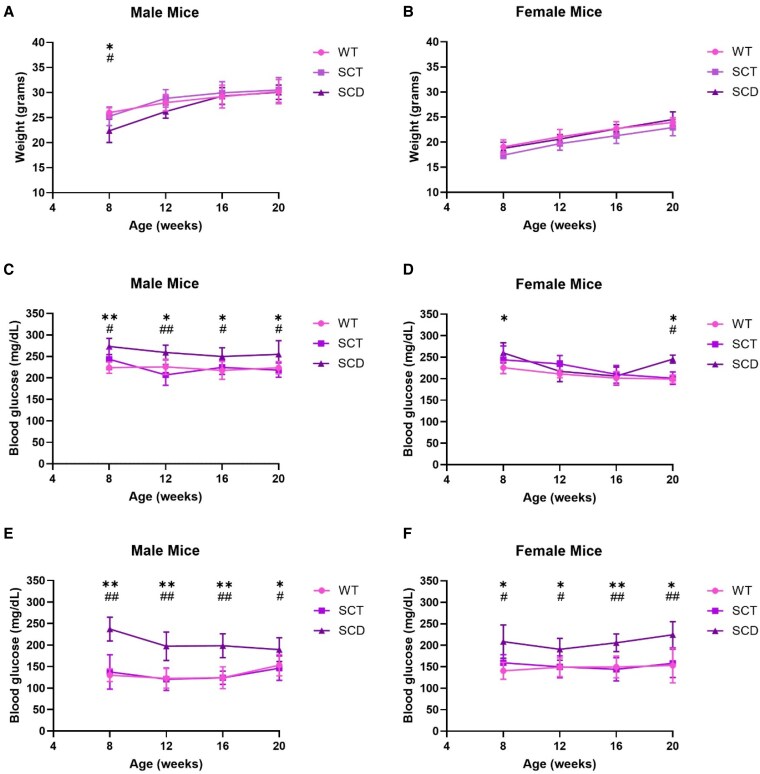
Compared with WT and SCT mice, SCD mice exhibit a similar longitudinal weight trend and greater ad libitum and fasting glucose levels. Normal chow-fed male (A, C, E) and female (B, D, F) Townes mice were assessed at age 8, 12, 16, and 20 weeks for weight trend (A, B), ad libitum blood glucose (C, D), and blood glucose level after an overnight 16-hour fast (E, F). All data are reported as the mean ± SD. Differences in weight or blood glucose were assessed at each time point with the Student *t* test, with *P* < .05 indicating significance. An asterisk (*) represents a significant statistical comparison between WT and SCD mice, *P < .05, **P < .01. A hashtag (#) represents a significant statistical comparison between SCT and SCD mice, #*P* < .05, ##*P* < .01. n = 4-8 mice per group.

The body weights of WT, SCT, and SCD male and female mice were assessed periodically (Fig. 2A and 2B). Male SCD mice weighed less than WT and SCT mice at 8 weeks of age but became comparable thereafter (Fig. 2A). This correlates with observations that male children and adolescents with SCD are at higher risk of being underweight (26, 27). Female WT, SCT, and SCD mice had comparable weight trends (Fig. 2B).

### SCD Mice Exhibit Greater Ad Libitum and Fasting Blood Glucose Levels Than SCT Mice and Littermate Controls

We next examined blood glucose during ad libitum feeding (Fig. 2C and 2D) and after an overnight fast (Fig. 2E and 2F). Male SCD mice exhibited greater ad libitum (Fig. 2C) and fasting (Fig. 2E) blood glucose than WT and SCT mice at all examined time points. Female SCD mice exhibited higher ad libitum blood glucose than WT mice at 8 weeks and WT and SCT mice at 20 weeks (Fig. 2D), as well as greater fasting blood glucose at all time points (Fig. 2F). In all cases, WT and SCT mice exhibited comparable blood glucose levels (Fig. 2C-2F). These data suggest that the presence of 2 copies of the HbS gene has a role in promoting glycemic dysregulation in male and female mice beginning at an early age and that the effect is not due to weight differences.

### SCD Mice Exhibit Declining Fasting Serum Insulin With Aging

**Figure 3.**
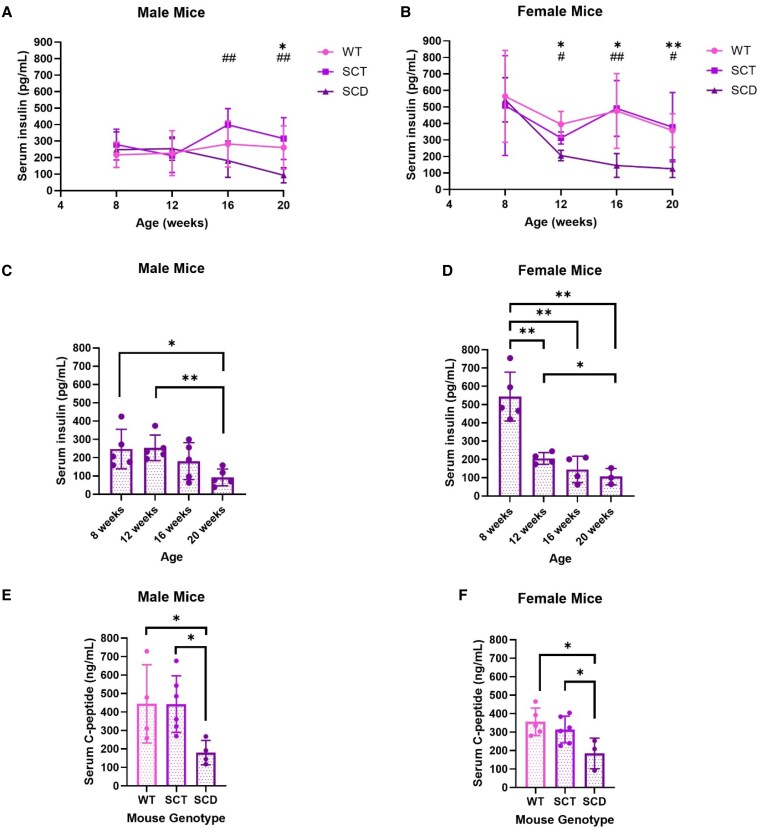
SCD mice exhibit declining fasting serum insulin levels with aging. After an overnight 16-hour fast, male (A, C, E) and female (B, D, F) Townes mice were analyzed for fasting serum insulin (A-D) and c-peptide levels (E, F) using tail vein blood followed by a mouse insulin and c-peptide ELISA, respectively. All data are reported as the mean ± SD, and differences in insulin levels between WT, SCT, and SCD mice groups were assessed with the Student *t* test, with *P* < .05 indicating significance. In A and B, an asterisk (*) represents a significant statistical comparison between WT and SCD mice, *P < .05, **P < .01. A hashtag (#) represents a significant statistical comparison between SCT and SCD mice, #*P* < .05, ##*P* < .01. In C-F, *P < .05, **P < .01. n = 4-7 mice.

Since SCD mice displayed greater ad libitum and fasting blood glucose levels from 8 to 20 weeks of age, we next assessed fasting insulin levels over this time period. Serum insulin level in SCD male mice was significantly lower than in sex-matched WT and SCT mice at 20 weeks of age compared to SCD males at 8 weeks of age (Fig. 3A), and was significantly lower in SCD female mice compared to sex-matched WT and SCT mice at 12, 16, and 20 weeks compared to SCD females at 8 weeks of age (Fig. 3B). Insulin levels in WT and SCT males and females were comparable. Analysis of SCD males (Fig. 3C) and females (Fig. 3D) shows declining fasting insulin with aging, predominately from weeks 12 to 20 in males and 8 to 12 weeks in females. Regardless of sex, SCD mice exhibited statistically lower fasting serum insulin at 20 weeks than their insulin level at 8 and 12 weeks of age. Correlating with lower fasting insulin at 20 weeks of age, male and female SCD mice exhibited lower serum c-peptide levels than age- and sex-matched WT and SCT mice (Fig. 3E and 3F). As ad libitum and fasting hyperglycemia (Fig. 2C-2F) precedes decreased serum insulin in SCD mice (Fig. 3A and 3B), these data suggest the possibility of progressive pancreatic β cell dysfunction in the presence of 2 copies of HbS.

### 20-Week-Old WT, SCT, and SCD Mice Exhibit Comparable Responses to Glucose and Insulin Loads

**Figure 4.**
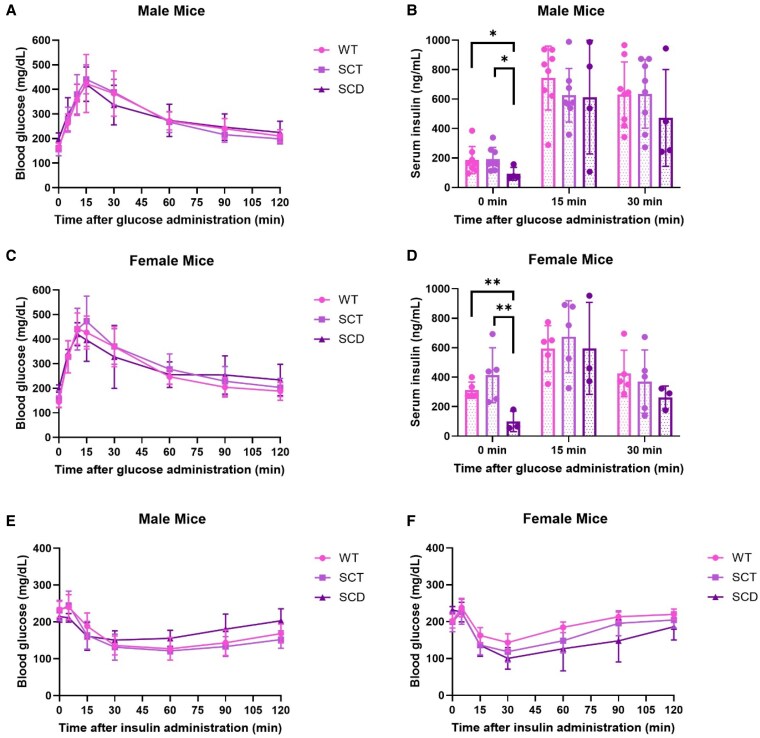
WT, SCT, and SCD Townes mice exhibit comparable post–glucose load glucose excursions and insulin tolerance. After a 16-hour overnight fast, 20-week-old male (A, B) and female (C, D) Townes mice were administered a 2 g/kg body weight glucose load by intraperitoneal injection and blood glucose (A, C) and serum insulin levels (B, D) were assessed and the time points are shown. To assess insulin sensitivity (E, F), male (E) and female (F) Townes mice were fasted for 6 hours, then administered 0.75 units/kg body weight Humalog insulin by intraperitoneal injection, and blood glucose levels were measured for 2 hours to assess the response. All data are reported as the mean ± SD. Changes in glucose excursions following a glucose load and insulin tolerance tests were analyzed by 2-way ANOVA, whereas serum insulin levels were assessed with a Student *t* test, with *P* < .05 indicating significance. Two-way ANOVA statistics: for glucose tolerance testing in males (A), WT vs SCT *P* = .929, WT vs SCD *P* = .841, SCT vs SCD *P* = .860; in females (C), WT vs SCT *P* = .580, WT vs SCD *P* = .765, SCT vs SCD *P* = .870; for insulin tolerance testing in males (E), WT vs SCT *P* = .359, WT vs SCD *P* = .635, SCT vs SCD *P* = .187; in females (F), WT vs SCT *P* = .104, WT vs SCD *P* = .087, SCT vs SCD *P* = .578. For serum insulin levels analyzed in B and D, *P < .05, **P < .01. n = 4-8 mice.

We next examined glucose and insulin tolerance by the intraperitoneal glucose tolerance test (IPGTT) and insulin challenge, respectively, at age 20 weeks (Fig. 4). In both males and females, WT, SCT, and SCD mice demonstrated comparable glucose excursions after a glucose load (Fig. 4A and 4C). SCD mice had lower fasting insulin levels than sex-matched WT and SCT mice at 0 minutes (Fig. 4B and 4D), correlating with the above findings (Fig. 3A and 3B), but had comparable insulin levels at 15 and 30 minutes into the IPGTT (Fig. 4B and 4D). All SCD mice started the IPGTT with higher blood glucose than WT and SCT mice, correlating with the findings above (Fig. 2E and 2F).

After a bolus of Humalog, insulin tolerance testing demonstrated no difference in insulin sensitivity between male (Fig. 4E) and female (Fig. 4F) WT, SCT, and SCD mice. These data suggest no overt alteration in insulin sensitivity in the presence of HbS and further indicate that pancreatic β cells may still be able to produce and/or secrete adequate insulin postprandially.

### SCD Mice Have Heavier Spleens and Livers Than Sex-Matched WT and SCT Mice

**Figure 5.**
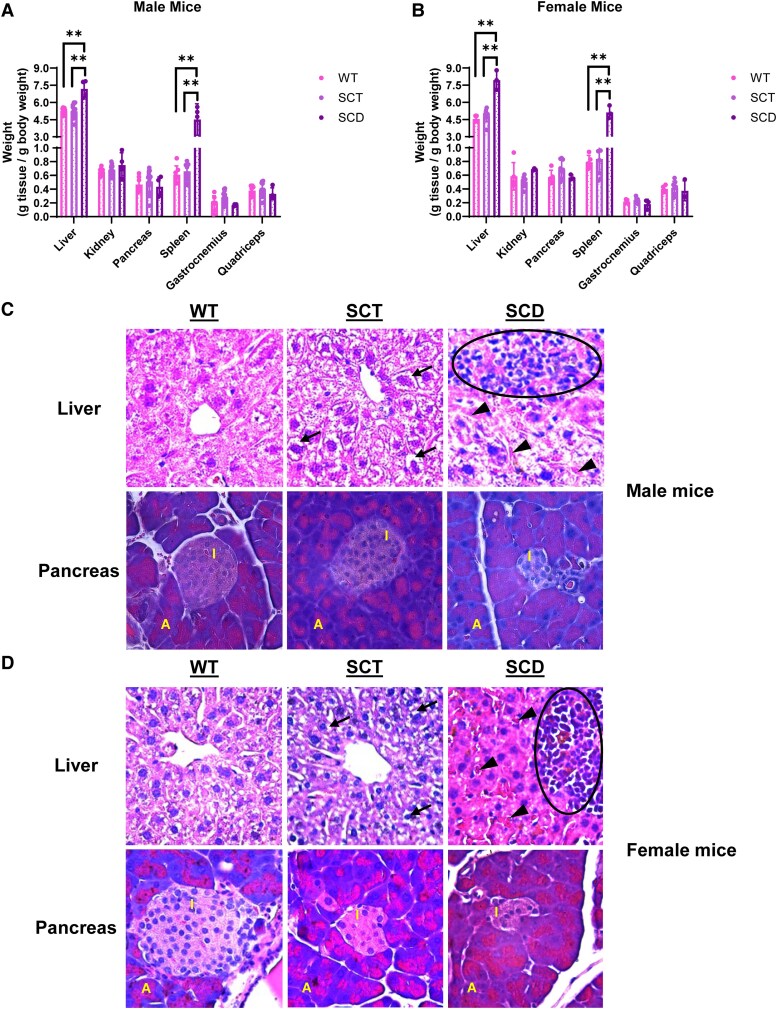
In addition to a larger spleen, SCD mice exhibit a larger liver than WT and SCT mice, with evidence of tissue injury but a morphologically comparable pancreas. The weights of the liver, kidney, pancreas, spleen, gastrocnemius muscle, and quadriceps muscle in male (A) and female (B) Townes mice were measured and normalized to body weight. Histopathological analyses of liver sections in WT, SCT, and SCD male (C) and female (D) Townes mice show vacuolization (arrows) in hepatocytes of SCT mice, as well as areas of hepatic necrosis with mononuclear infiltrates (oval region) and vascular congestion (arrowheads) in SCD mice. Histopathological analyses of pancreas sections in WT, SCT, and SCD mice demonstrate comparable overall architecture with clearly defined islet of Langerhans (“I”) and acinar cells (“A”). Tissue weight data are reported as the mean ± SD and were analyzed by the Student *t* test, with *P* < .05 signifying significance; **P < .01. Histopathological assessments of the liver and pancreas were performed using a light microscope, 40× objective. n = 3-8 mice for weight analysis; n = 3 mice for histopathological analyses.

We next assessed the weights of tissues involved in the regulation of whole-body glucose homeostasis, including skeletal muscle, liver, kidney, and pancreas (28, 29), as well as the spleen as a pathognomonic indicator for SCD in this mouse model (30, 31). Normalizing tissue weight to body weight, we found that SCD mice had heavier livers than sex-matched WT and SCT mice (Fig. 5A and 5B). Kidney, pancreas, gastrocnemius, and quadriceps weights were comparable. Expectedly, the spleens of male and female SCD mice weighed substantially more than that of WT and SCT mice.

### Immunohistochemical Analysis of the Liver and Pancreas From WT, SCT, and SCD Mice

With the findings of greater liver weight (Fig. 5A and 5B) and reduced fasting serum insulin with aging (Fig. 3C and 3D) in SCD mice, we next examined liver and pancreas morphology and iron deposition. Compared with WT mice (Fig. 5C and 5D, upper left panels), the livers of male and female SCT mice demonstrated hepatocyte vacuolization (Fig. 5C and 5D, upper middle panels, arrows), suggestive of mild subacute hepatocyte injury, though the overall architecture appeared to be maintained (32). Compared with WT mice, SCD mice livers demonstrated areas of necrosis with mononuclear infiltration (Fig. 5C and 5D, upper right panels, oval), areas of vascular congestion (Fig. 5C and 5D, upper right panels, arrowheads), and disruption of the overall architecture. The pancreas of male and female WT, SCT, and SCD mice (Fig. 5C and 5D, bottom panels) demonstrates traditional pancreatic architecture with islets of Langerhans (Fig. 5C and 5D, label “I”) embedded within exocrine acinar cells (Fig. 5C and 5D, label “A”).

**Figure 6.**
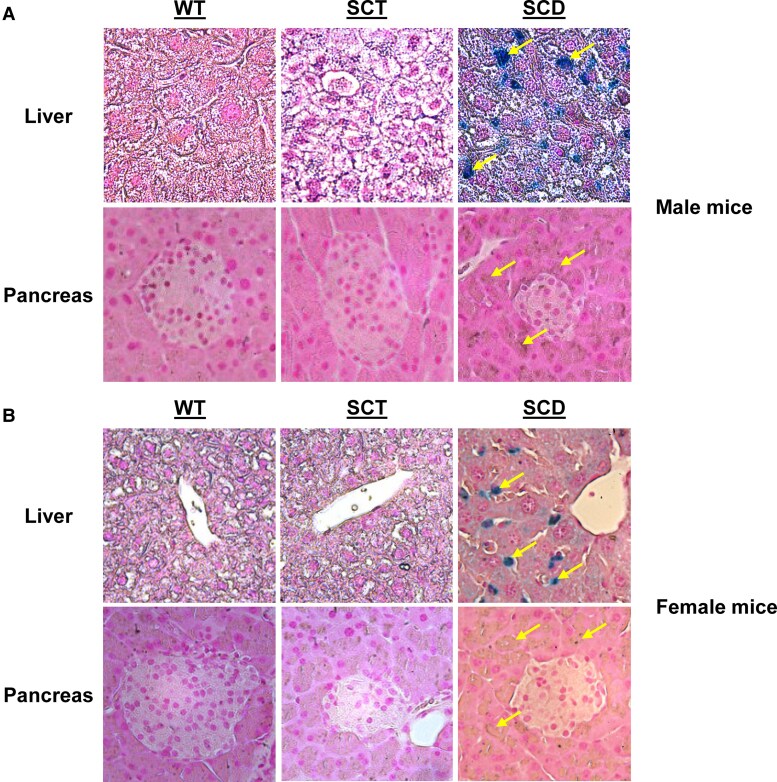
SCD mice exhibit iron deposition within the liver and the exocrine pancreas. Sections of liver and pancreas in male (A) and female (B) Townes mice were analyzed for iron deposition with Prussian blue staining. Staining was noted in SCD mice livers and within the pancreatic acinar cells (arrows), but not within the islets of Langerhans. Assessments of liver and pancreas were performed using a light microscope, 40× objective. n = 3 mice.

Chronic hemolysis in SCD results in increased demand for iron to support erythropoiesis, which, over time, leads to secondary iron deposition in various tissues, tissue injury, and organ failure (33, 34). We next assessed iron deposition in the liver and pancreas (Fig. 6). Within the liver, male and female SCD mice exhibited iron deposition (Fig. 6A and 6B, upper right panels, arrows), which was not seen in WT and SCT mice (Fig. 6A and 6B, upper left and center panels). Within the pancreas, WT and SCT mice did not demonstrate iron deposition (Fig. 6A and 6B, lower left and center panels). In SCD mice, iron deposition was noted in the pancreatic acinar cells (Fig. 6A and 6B, lower right panels, arrows), but, interestingly, there was no iron deposition visualized within the islet of Langerhans.

These findings suggest that increased liver weight found in SCD mice may be partly due to traditional pathophysiological changes seen in this organ in SCD, including necrosis, inflammation, congestion, and iron deposition. Furthermore, the finding of generally maintained pancreatic architecture, and lack of iron deposition in the pancreatic islets of SCD mice may suggest that pancreatic insulin dynamics are altered through non-iron–mediated biochemical mechanisms.

### Male SCD Mice Exhibit Lower Pancreatic Insulin Content, Whereas Male and Female SCT and SCD Mice Have Reduced Islet Size and β Cell Mass

**Figure 7.**
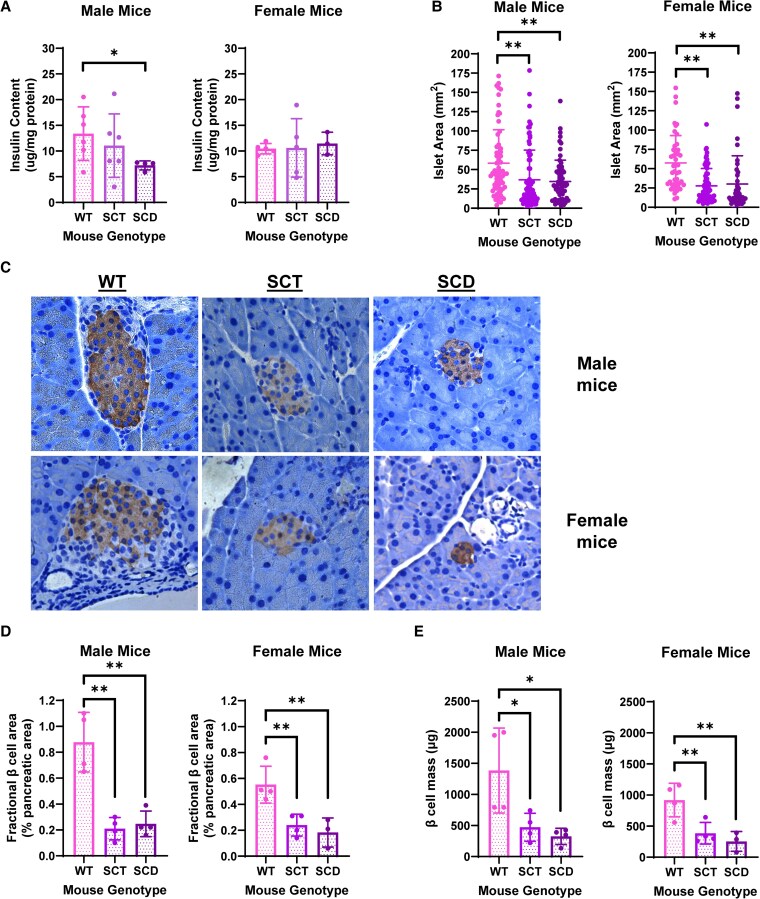
Compared with sex-matched WT mice, male SCT and SCD mice exhibit declining total pancreatic insulin content, and male and female SCT and SCD mice exhibit decreased pancreatic islet area, fractional β cell area, and β cell mass. The pancreatic tail region from male and female Townes mice was homogenized in acid–ethanol solution and centrifuged, and insulin content was assayed from the supernatant using an ELISA and normalized to total protein content (A). Pancreatic islets from male and female WT, SCT, and SCD Townes mice (4 mice per group, 30 pancreatic sections per mouse) were identified and measured for surface area (B). A representative islet stained for insulin is shown for each genotype from male and female Townes mice in C. Approximately 32-36 sections of the pancreatic head from male and female Townes mice were stained for insulin, and the fractional β cell area (D) (percent of total insulin-stained area per pancreatic section) and β cell mass (E) were calculated. Data in A, B, D, and E are reported as the mean ± SD and were analyzed by the Student *t* test, with *P* < .05 indicating significance, *P < .05, **P < .01. Imaging of pancreatic sections was performed using a light microscope, 40× objective. n = 3-6 mice per group for pancreatic insulin content; n = 4 mice per group for islet area, fractional β cell area, and β cell mass.

With our findings of ad libitum and fasting hyperglycemia, coupled with decreased fasting insulin and c-peptide in SCD mice at 20 weeks of age, suggestive of developing pancreatic β cell dysfunction, we next assessed pancreatic insulin content (Fig. 7A). In males, total pancreatic insulin content was decreased in SCD mice and trended down in SCT mice compared with WT mice. Contrary to this finding, female WT, SCT, and SCD mice exhibited similar pancreatic insulin content. We next assessed the morphological size of the islet of Langerhans and β cell mass. Assessment of the surface area of individual islets (Fig. 7B) demonstrated that SCT and SCD mice had overall smaller islets than sex-matched WT mice. Representative images of the pancreatic islets are shown in Fig. 7C, showing SCT and SCD males and females with overall smaller islets and, as such, a smaller area occupied by β cells. In addition to smaller islets, SCT and SCD mice also had significantly less insulin staining per section than sex-matched WT mice (Fig. 7D), as well as significantly decreased β cell mass (Fig. 7E). These data suggest that with aging, there is a reduction in ability to synthesize, process, and/or secrete insulin in SCD mice, which may be compensated for in SCT mice in order to produce and secrete insulin adequately to maintain euglycemia.

## Discussion

For the reasons noted above, including the need to accurately diagnose diabetes (1-4), the rising prevalence of diabetes in individuals with HbS, and the increased risk for diabetes-related complications in those with HbS (15-19), it is critical to understand how HbS is linked to glycemic regulation. Therefore, we utilized a mouse model of human SCD to dissect how HbS affects glucose homeostasis. We found that normal chow-fed SCD mice exhibited higher ad libitum and fasting blood glucose than WT and SCT mice from 8 to 20 weeks of age, without differences in weight trend, glucose, or insulin tolerance. Furthermore, SCD mice exhibited declining fasting insulin levels with aging and were found to have smaller pancreatic islets and significantly lower β cell mass than WT mice. Notably, islet size and β cell mass of SCT mice were similar to SCD mice regardless of sex, though the former displayed blood glucose and serum insulin similar to WT mice. These findings demonstrate that the presence of 2 copies of HbS negatively impacts glucose regulation from an early age and can promote the development of pancreatic β cell dysfunction with aging. Further, these findings exemplify the importance of close glycemic monitoring in individuals with SCD, who can be at high risk of developing diabetes in the absence of an obesogenic state.

A key observation in the data above is that hyperglycemia develops early in SCD mice before reductions in fasting serum insulin. This suggests that the presence of 2 copies of HbS promotes early hyperglycemia, both in fasting and feeding states (Fig. 2C-2F), potentially independent of the endocrine pancreas. As hyperglycemia becomes chronic, we noted the development of pancreatic β cell dysfunction with aging. The mechanism by which HbS leads to elevated blood glucose levels with normal serum insulin is unclear. Some possibilities are that SCD is characterized by chronic oxidative stress due to higher levels of reactive oxygen species (ROS), which accumulate through multiple mechanisms, including HbS autoxidation, hemolysis, endothelial dysfunction, inflammation, and ischemia–reperfusion injury stemming from repetitive vaso-occlusion (35-37). Increased ROS production is implicated in developing insulin resistance and hyperglycemia (38, 39). Though we found no difference in insulin sensitivity between WT, SCT, and SCD mice at 20 weeks of age (Fig. 4E and 4F), glucose disposal can be subject to counterregulatory effects (40). Assessments of insulin signaling pathway activity in critical glucose-handling organs, such as the liver, which shows pathological findings in SCD mice, will help discern if there are changes in insulin sensitivity at the tissue level. Furthermore, gold-standard insulin clamp studies have been utilized before to assess insulin resistance in the presence of ROS (41). Assessments of ROS in conjunction with hyperinsulinemic–euglycemic clamp studies in younger Townes mice exhibiting hyperglycemia but comparable fasting insulin levels are essential to dissect differences in insulin sensitivity in Townes mice and the role ROS may have in the process.

A second possible mechanism by which HbS may cause early hyperglycemia involves increased iron storage. Excess iron in SCD tends to be stored in the liver, pancreas, and myocardium, which can lead to organ dysfunction (34). Here, we demonstrated iron deposition in the liver and exocrine pancreas of SCD mice (Fig. 6). Increases in body iron in SCD have been linked to increased oxidative stress and insulin resistance, which at the level of the liver, can interfere with insulin metabolism and negatively impact insulin signaling, hence leading to hyperglycemia (38, 42). Though we did not see iron deposition in the pancreatic islets, there was iron in the surrounding acinar cells, which may promote oxidative stress, inflammation, and tissue damage. Assessment of ferritin levels in young Townes mice is warranted to understand the impact of iron stores on the development of early hyperglycemia.

Contrasting with the above possibilities, a third potential mechanism is that β cell dysfunction is present in 8-week-old SCD mice. Since there is hyperglycemia in the fasting and fed states in SCD mice compared with sex-matched WT and SCT mice at this young age, it would be expected that serum insulin levels should be greater in SCD mice. However, fasting insulin levels are comparable regardless of genotype at 8 weeks. These data raise the possibility that the production of insulin by pancreatic β cells is insufficient to curb hyperglycemia. A thorough assessment of pancreatic islets and β cell function at 8 weeks of age is warranted to test the hypothesis of insufficient insulin production leading to early hyperglycemia.

Understanding β cell function at 8 weeks is important to understanding the development of pancreatic islet dysfunction as SCD mice age. We found that SCD mice have smaller pancreatic islets and lower β cell mass at 20 weeks of age than WT mice. This raises the question as to the morphology of pancreatic islets when SCD mice are 8 weeks of age when we first noted severe hyperglycemia. If the islets are similar in size and β cell mass to WT mice, then our 20-week findings may be due to chronic hyperglycemia resulting in β cell demise and reduced β cell mass that cannot deliver adequate insulin (43, 44). Alternatively, or in addition to chronic hyperglycemia, repeated sickling and iron deposition may lead to the development of common bile duct stones and repeated bouts of pancreatitis and peripancreatic inflammation, which may further affect the function of pancreatic islets (45-47). On the other hand, if SCD mice have smaller islets and decreased β cell mass at 8 weeks of age compared with WT mice, then β cells from SCD mice would be expected to have enhanced insulin synthesis and/or secretion in order to produce insulin at levels similar to WT mice.

Vasoconstriction and vaso-occlusion of blood vessels in SCD due to erythrocyte sickling can also alter the blood flow to islets of Langerhans, leading to alteration in insulin dynamics over time. The islet of Langerhans, despite making up only 2% of the pancreas, receives 10-fold as much blood per volume via arterioles as the acinar cells (48). Blood flow to the islets varies, with about 66% of islets having blood flow following a center to periphery pattern serving the centrally located β cells first, and 34% of islets having blood flow in a periphery to center pattern serving peripherally located α cells first (49, 50). Medical conditions such as diabetes and cancer have been previously shown to alter the β to α cell ratio and resultant islet paracrine signaling (51-53). A closer assessment of the extent of pancreatic sickling and blood flow abnormalities in SCT and SCD mice, as well as an analysis of islet cell composition in these mice, may further provide mechanistic insights into the link between HbS and the glycemic regulatory capabilities of pancreatic islets.

Interestingly, SCT mice have reduced pancreatic islet size, fractional β cell area, and β cell mass compared with WT mice at 20 weeks of age but have comparable blood glucose and insulin levels at all examined time points. This may suggest the presence of compensatory mechanisms in SCT that maintain euglycemia, such as enhanced β cell insulin production and secretion. The liver of SCT mice demonstrates hepatocyte vacuolization, which is an early sign of liver injury (32). Also, as discussed above, the prevalence of T2DM is greater in US service members with SCT than in those without, and SCT, along with SCD, has been shown to increase the risk for diabetic complications (14-18). These observations emphasize that having 1 copy of the sickle β globin gene is not an entirely benign condition. Assessing SCT mice at older ages is needed to determine if SCT mice eventually lose compensatory abilities, leading to β cell demise and glycemic dysregulation.

Compared with WT male mice, SCD mice exhibited declining serum insulin with aging in conjunction with decreased pancreatic insulin content, islet size, and β cell mass at 20 weeks of age. This combination of findings suggests a role of HbS in β cell demise, leading to inadequate pancreatic insulin production. In female mice, contrary to males, SCD mice exhibited pancreatic insulin content similar to WT mice despite having reduced serum insulin with aging and decreased islet size and β cell mass. Studies have demonstrated that males tend to have more severe complications of SCD and diabetes than females (54, 55). Further phenotypical analyses of the pancreas and insulin dynamics in males and females over time are warranted to determine the precise timeline and mechanisms leading to pancreatic β cell dysfunction.

In conclusion, this work is an initial characterization of the glycemic phenotype of WT, SCT, and SCD Townes mice, providing an initial understanding of how HbS affects glucose homeostasis. We have observed here that 2 copies of the HbS gene promote early onset hyperglycemia, seen as early as 8 weeks of age—the first analysis time point—in the presence of comparable fasting insulin levels as SCT and littermate control mice. Hyperglycemia in the fasting and feeding states persist as SCD mice age and becomes accompanied by decreasing fasting serum insulin with aging and reduced islet size and β cell mass, suggesting developing pancreatic β cell dysfunction. These findings are significant because they demonstrate the early onset of hyperglycemia in SCD and enhanced risk of progression to clinical diabetes. Studies aimed at further elucidating the precise biochemical mechanisms by which HbS affects glucose homeostasis and pancreatic insulin dynamics will be critical to (1) understand how to identify and monitor glycemia in patients with HbS accurately, (2) identify potential target sites for new therapeutics, and (3) determine which current treatment options would benefit patients with SCT/SCD the most.

## Data Availability

Some or all datasets generated during and/or analyzed during the current study are not publicly available but are available from the corresponding author on reasonable request.

## Prior Presentation

A portion of the data in Figures 2, 3, and 4 was accepted as an abstract and poster presentation at the 2024 Endocrine Society Annual Meeting, June 1-4, 2024, Boston, MA.

## Abbreviations

ANOVA: analysis of variance
ELISA: enzyme-linked immunosorbent assay
HbA1c: hemoglobin A1c
HbS: hemoglobin S
IPGTT: intraperitoneal glucose tolerance test
OCT: optimal cutting temperature
ROS: reactive oxygen species
SCD: sickle cell disease
SCT: sickle cell trait
T2DM: type 2 diabetes mellitus
WT: wild type
